# Multiple Sclerosis: Evaluation of Purine Nucleotide Metabolism in Central Nervous System in Association with Serum Levels of Selected Fat-Soluble Antioxidants

**DOI:** 10.1155/2014/759808

**Published:** 2014-05-06

**Authors:** Ľubomír Kuračka, Terézia Kalnovičová, Jarmila Kucharská, Peter Turčáni

**Affiliations:** ^1^Institute of Medical Chemistry, Biochemistry and Clinical Biochemistry, Faculty of Medicine, Comenius University, 811 08 Bratislava, Slovakia; ^2^1st Department of Neurology, Faculty of Medicine, Comenius University, 813 69 Bratislava, Slovakia; ^3^Pharmacobiochemical Laboratory of 3rd Medical Clinic, Faculty of Medicine, Comenius University, 811 08 Bratislava, Slovakia

## Abstract

In the pathogenesis of demyelinating diseases including multiple sclerosis (MS) an important role is played by oxidative stress. Increased energy requirements during remyelination of axons and mitochondria failure is one of the causes of axonal degeneration and disability in MS. In this context, we analyzed to what extent the increase in purine catabolism is associated with selected blood lipophilic antioxidants and if there is any association with alterations in serum levels of coenzyme Q_10_. Blood serum and cerebrospinal fluid (CSF) samples from 42 patients with diagnosed MS and 34 noninflammatory neurologic patients (control group) were analyzed. Compared to control group, MS patients had significantly elevated values of all purine nucleotide metabolites, except adenosine. Serum lipophilic antioxidants **γ**-tocopherol, **β**-carotene, and coenzyme Q_10_ for the vast majority of MS patients were deficient or moved within the border of lower physiological values. Serum levels of TBARS, marker of lipid peroxidation, were increased by 81% in the MS patients. The results indicate that the deficit of lipophilic antioxidants in blood of MS patients may have a negative impact on bioenergetics of reparative remyelinating processes and promote neurodegeneration.

## 1. Introduction

Multiple sclerosis (MS) is an inflammatory immune mediated demyelinating disease of the central nervous system (CNS). An energy deficient state and oxidative and nitrative stress have been implicated in the degeneration of axons in multiple sclerosis [1–4]. In chronic lesions, axonal degeneration correlates with the extent of inflammation and leads to axonal loss through a slow burning process [5].

Mickel [6] proposed that a lipid peroxidation disturbance caused by free radicals production is involved in the breakdown of the myelin sheath. Since then several studies have demonstrated the role of increased free radical production and/or a decreased antioxidant defense in CNS as causal factors of MS [1, 4, 7, 8].

Following demyelination the axonal membrane undergoes a number of changes including an increase in number of sodium channel within demyelinated part of the axon [9–11]. The maintenance of intra-axonal ionic balance and resting membrane potential following the influx of sodium through the increased sodium channels relies on the largest consumer energy in the central nervous system, Na^+^/K^+^ ATPase [12]. In noninflammatory environments this increase in energy demand of axons lacking a healthy myelin sheath is apparent by the changes in density and activity of energy producing organelles mitochondria, the most efficient producers of energy [2, 13, 14].

Though the principal site of MS pathology is the CNS, the lipid status and the membrane properties in the platelets and erythrocytes in the peripheral blood are also altered [7]. Increased lipid peroxide levels have been observed both in the cerebrospinal fluid and in the blood of MS patients [7, 8, 15]. Lack of sufficient vitamin A and E in the diet has been suggested to be a risk factor for the onset of the disease [7, 16]. However, other studies have found that the plasma levels of these vitamins are similar in MS patients and in controls [7, 17].

The present study examined serum levels of vitamin A (*β*-carotene), vitamin E (*γ*- and *α*-tocopherol) isomers, and coenzyme Q_10_ (antioxidant, which is an indicator of bioenergetic state) in MS patients in relation to the cerebrospinal fluid levels of purine nucleotides degradation products (adenosine, inosine, hypoxanthine, xanthine, and uric acid), which are known to be produced during energy deficiency. Furthermore, we were interested to discover which of these lipophilic antioxidants is associated the most with lipid peroxidation and degradation of purine nucleotides in MS patients.

## 2. Materials and Methods

### 2.1. Clinical Evaluation of the Patients and Preparation of Samples

Blood serum and cerebrospinal fluid (CSF) samples from 42 patients diagnosed with multiple sclerosis (MS) according to McDonald's rule were analyzed. Test group consisted of 32 females and 10 males with average age of 36.3 ± 11.97 years. Each patient had relapsing-remitting form and was out of relapse at the time. According to our knowledge, patients were not presented with any other serious illnesses. The control group was created from neurological patients with noninflammatory diseases of the central nervous system (*n* = 34, 8 males, 26 females) with an average age of 36.06 ± 11.92 years, who had routine CSF analysis and biochemical parameters within the physiological values. Each proband signed informed consent and agreed with the investigation of mentioned parameters. Ethical committee statement was not necessary since the examination of parameters was indicated by neurologist and was a component of diagnostic process. None of the patients had demyelinating disease or any other diseases associated with an increase of oxidative stress and degradation of purine nucleotides. Blood and CSF samples were taken at the same time. Aliquots of CSF and serum samples were centrifuged, coded, and immediately stored at −70°C in polypropylene tubes until being assayed.

### 2.2. Laboratory Assays

CSF levels of uric acid (UA), hypoxanthine (Hyp), xanthine (Xan), inosine (Ino), and adenosine (Ado) were measured by isocratic HPLC method (high-performance liquid chromatography) with UV detection (254 nm). Deproteinized and centrifuged samples were loaded (20 *μ*L) onto a LiChroCART 250-4 column filled with LiChrospher 100 RP-18 (5 *μ*m) (Merck, Germany). The chromatographic column was connected to an HPLC apparatus consisting of a LaChrom L-7100 pump system and L-7400 UV detector (Merck-Hitachi, Germany). Data acquisition and analysis were performed by a PC using the CSW v1.7, (DataApex Ltd., Czech). Mobile phase included 60 mmol/L KH_2_PO_4_, 2% methanol (v/v), pH 2.9.

Serum *α*- and *γ*-tocopherol (*α*T and *γ*T), *β*-carotene (*β*C), and coenzyme Q_10_ (CoQ_10_) were measured by modified isocratic HPLC method with UV detection at 275 nm (CoQ_10_), 295 nm (tocopherols), and 450 nm (*β*-carotene) [18, 19].

Serum levels of thiobarbituric acid reactive substances (TBARS) were determined spectrophotometrically at 532 nm according to Janero and Burghardt [20].

State of blood-brain barrier (BBB) of patients was evaluated via QA-index, as ratio between albumin concentration in CSF and in blood serum multiplied by 1000. QA-index higher than 7.4 indicates BBB deterioration.

Intrathecal IgG production was evaluated via IgG index (IgG index = IgG_CSF_/albumin_CSF_ : IgG_s_/albumin_s_) and Reiber's index (RIG) [21].

### 2.3. Statistical Analysis

All statistical analyses were carried out using StatsDirect statistical software, version 2.7.2 (StatsDirect Sales, Sale, Cheshire M33 3UY, United Kingdom). *P* < 0.05 was considered significant. For normal distribution of data, the means and standard deviations were shown and Student's *t*-test for comparing two independent samples was used. For non-Gaussian distribution, the median values and 25–75% interquartile range (IQR) were shown and independent variables were compared using the nonparametric Mann-Whitney *U* test. The linear relationship between continuous variables was evaluated using the Spearman's correlation coefficient.

## 3. Results

Selected basic biochemical parameters in the serum and cerebrospinal fluid (CSF) are shown in Table 1 characterizing the control group and the group of patients with multiple sclerosis (MS) in comparison to the reference values. The group with multiple sclerosis patients (*n* = 42, the average age of 36.49 ± 11.97 years) was characterized by pathologically high CSF levels of immunoglobulin IgG, with evidence of increased intrathecal synthesis of immunoglobulin IgG expressed by elevated values of IgG index and Reiber's index (RIG) (Table 1). Average QA values of MS patients (that characterize the integrity of blood-brain barrier) were within the physiological range but in comparison to controls were significantly higher (*P* = 0.0293).

### 3.1. Purine Nucleotide Degradation Products

In Table 2 are shown CSF levels of purine nucleotide degradation products adenosine (Ado), inosine (Ino), hypoxanthine (Hyp), xanthine (Xan), and uric acid (UA) in comparison with a control set of neurological patients with noninflammatory CNS diseases. Compared to control group, MS patients had a significantly elevated values of all purine nucleotide metabolites, except of adenosine, which were significantly lower (0.20 ± 0.16 *μ*mol/L versus 0.44 ± 0.20 *μ*mol/L) (Table 2).

### 3.2. Serum Lipophilic Antioxidants and TBARS

Serum lipophilic antioxidants *γ*-tocopherol (*γ*T), *α*-tocopherol (*α*T), the ratio of *γ*/*α*-tocopherol (*γ*/*α*T), *β*-carotene (*β*C), and coenzyme Q_10_ (CoQ_10_) in the vast majority of cases were lower than normal or were in the close proximity to the bottom border of the physiological values (Table 3).

**Figure 1:**
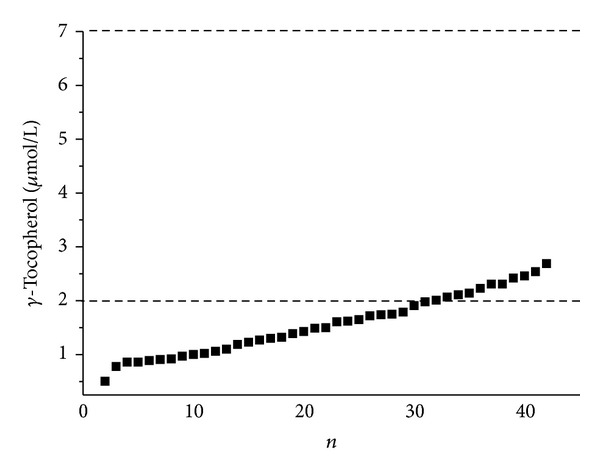
Serum *γ*-tocopherol levels in multiple sclerosis patients (*n* = 42) compared to reference range (2–7 *μ*mol/L).

The greatest incidence of antioxidants deficiency occurred at *γ*T levels, where up to 71.4% of MS patients had subliminal serum values of *γ*T and *γ*T values of the rest of the MS patients were in the bottom border of the *γ*T physiological values (Figure 1). The ratio between the isomers of vitamin E, *γ*- and *α*-tocopherols (*γ*/*α*T), was reduced compared to the reference values of all analyzed MS patients. Serum *α*-tocopherol levels were within the borders of physiological values (Table 3). We observed deficit status in serum levels of *β*-carotene in approximately 30% and in CoQ_10_ levels in 45% of MS patients (Table 3).

**Figure 2:**
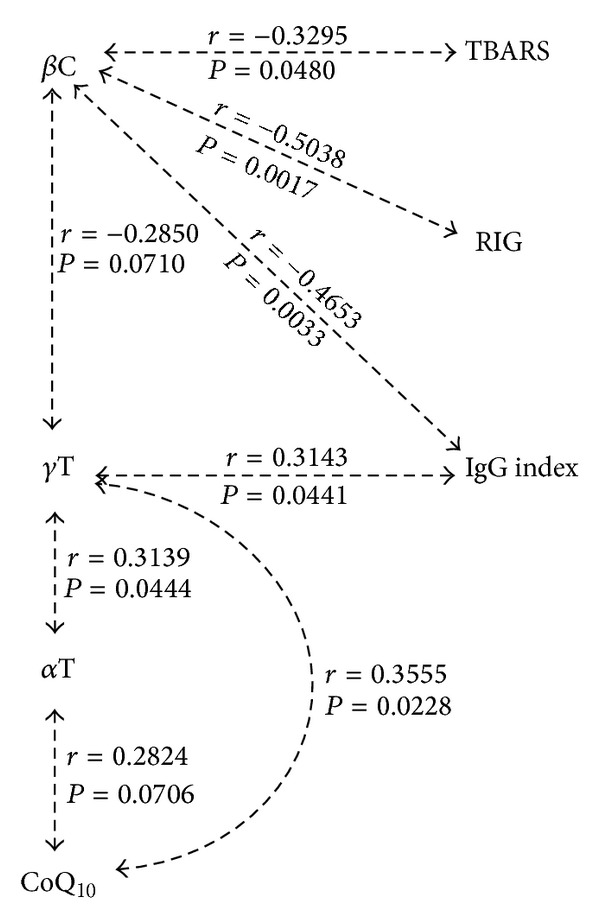
Spearman rank correlations among lipophilic antioxidants, TBARS, and intrathecal synthesis of IgG expressed by IgG index and Reiber's index. *β*C: *β*-carotene, *γ*T: *γ*-tocopherol, *α*T: *α*-tocopherol, CoQ_10_: coenzyme Q_10_, and TBARS: thiobarbituric acid reactive substances.

Serum levels of TBARS (thiobarbituric acid reactive substances) and marker of lipid peroxidation were increased by 81% in the MS patients (Table 3). These values correlated negatively with serum levels of *β*-carotene (*r* = −0.329; *P* = 0.048) (Figure 2). In addition, *β*-carotene negatively correlated with IgG index (*r* = −0.465, *P* = 0.003) and RIG (*r* = −0.504, *P* = 0.0017) (Figure 2).

### 3.3. Spearman Rank Correlations among Analyzed Parameters

Spearman rank correlations among lipophilic antioxidants and intrathecal synthesis of IgG expressed by IgG index and Reiber's index RIG (Figure 2) showed also significant relationship between CoQ_10_ and *γ*T (*r* = 0.355, *P* = 0.02) and between *γ*T and IgG index (*r* = 0.314, *P* = 0.04). Serum levels of *γ*T significantly correlated with levels of *α*-tocopherol as well (*r* = 0.314, *P* = 0.04) (Figure 2).

**Figure 3:**
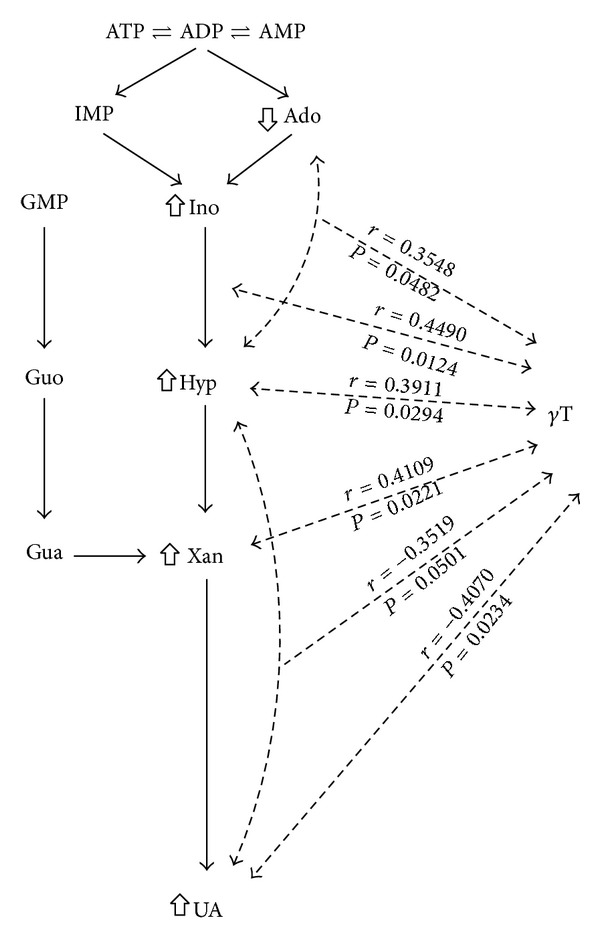
Spearman rank correlations between CSF purine nucleotide degradation products and serum *γ*-tocopherol in patients with multiple sclerosis. *γ*T: *γ*-tocopherol, Ado: adenosine, Ino: inosine, Hyp: hypoxanthine, Xan: xanthine, UA: uric acid, Guo: guanosine, and Gua: guanine.

Out of the measured antioxidants, *γ*-tocopherol correlated the most with CSF levels of purine nucleotide degradation products (Figure 3). Serum levels of *γ*T correlated positively with CSF levels of adenosine (*r* = 0.355, *P* = 0.05), metabolic turnover of adenosine to inosine (*r* = 0.449, *P* = 0.01), CSF levels of hypoxanthine (*r* = 0.391, *P* = 0.03), and xanthine (*r* = 0.411, *P* = 0.02) and negatively with CSF levels of uric acid (*r* = −0.407, *P* = 0.02) and metabolic turnover of hypoxanthine to uric acid (*r* = −0.352, *P* = 0.05) (Figure 3).

### 3.4. Purine Nucleotide Degradation Depending on the Serum Lipophilic Antioxidants Concentrations

With regard to the occurrence of not uniform serum deficit of lipophilic antioxidants MS patients, we analyzed our set of patients divided into 4 subgroups (Table 4(a)). The first group consisted of patients with deficiency of *γ*-tocopherol only (−*γ*T, *n* = 16). The second group consisted of patients with deficiency and thresholds to be deficit *γ*T and CoQ_10_ (−*γ*T*β*CoQ_10_; *n* = 16), the third group consisted of patients with deficiency and thresholds to be deficit *γ*T and *β*C (−*γ*T*β*C; *n* = 10), and the fourth group consisted of patients with deficiency and thresholds to be deficit *γ*T, *β*C, and CoQ_10_ (−*γ*TCoQ_10_ *β*C; *n* = 10). In accordance with the correlation relationships (Figure 3), the groups of MS patients with deficiency of *β*-carotene (−*γ*T*β*C and −*γ*TCoQ_10_ *β*C) had higher values of TBARS and higher intrathecal synthesis of IgG (IgGindex, RIG) compared to groups −*γ*T and −*γ*TCoQ_10_ (Table 4(a)).

Serum levels of CoQ_10_ in MS patients significantly interfere with the metabolism of purine nucleotides in CSF (Table 4(b)). CoQ_10_ deficiency leads to increased degradation of adenosine to inosine (Ino/Ado), which is manifested by significantly lower CSF levels of adenosine (group 2; Ado = 0.12 (0.07–0.17) *μ*mol/L) in comparison to MS patients with *γ*T deficiency (group 1; Ado = 0.17 (0.13–0.54) *μ*mol/L, *P* = 0.028).

All MS subgroups had significantly lower CSF levels of adenosine and significantly increased degradation of adenosine to inosine (Ino/Ado) and to hypoxanthine (Hyp/Ado) compared to the control group (Table 4(b)).

The group of MS patients with *β*-carotene deficiency had the increased degradation of the hypoxanthine to the xanthine (Xan/Hyp), as well as significantly higher CSF levels of uric acid compared to the control group (20.09 (17.88–27.54) versus 15.07 (14.12–16.02) *μ*mol/L, *P* = 0.024). In comparison to the control group, significantly elevated CSF levels of xanthine were observed in patients with *γ*T deficiency and *γ*T + CoQ_10_ deficiency only (Table 4(b)).

## 4. Discussion

The results presented suggest that, in patients with multiple sclerosis (MS) alteration in the metabolism of purine nucleotides (Table 2), reduced antioxidant and neuroprotection (Table 3) occur, and is associated with increased intrathecal synthesis of IgG. Similar results were reported by multiple authors [8, 22–25].

MS patients may suffer from a cell energy metabolism deficit that can be documented in biological fluids (cerebrospinal fluid and serum). The profile of compounds directly (CSF adenosine, inosine, hypoxanthine, xanthine, and uric acid) or indirectly (serum coenzyme Q_10_) reflects the imbalance between adenosine triphosphate (ATP) production and consumption. Increased purine nucleotide degradation observed in our MS patients (Table 2) is activated in situations that are associated with a decrease in the amount of ATP and the related rise of adenosine monophosphate (AMP) levels. AMP can be metabolized in the cells in two ways: (a) by deamination to inosine monophosphate (IMP) followed by dephosphorylation of IMP to inosine or (b) by dephosphorylation to adenosine and its deamination to inosine (Figure 3). At physiological concentrations of ATP deamination of AMP to IMP is preferred [26].

In experimental autoimmune encephalomyelitis (EAE), the animal model of MS, it has been effectively shown that efficiency in neuronal ATP biosynthesis is decreased due to mitochondrial malfunctioning, leading to cell energy state imbalance [27, 28]. If this occurs in MS patients too, a continuous outflow of ATP catabolites, including uric acid and its precursors, is expected from cerebral tissue of the MS lesions into the extracellular space. The picture of an energetic alteration in MS patients indirectly is reinforced by data referring to the coenzyme Q_10_ decrease in serum of MS patients (Table 3).

Coenzyme Q_10_ is essential for the energy production of the cells as an electron transporter in the mitochondrial respiratory chain. In the process of oxidative phosphorylation in mitochondria Coenzyme Q_10_ transfers electrons from complex I (NADH CoQ reductase) to complex III (cytochrome bc1 complex) or complex II (succinate dehydrogenase) on the complex III [29]. CoQ_10_ deficiency during the time of increased energy requirements causes a decrease in production of ATP and activates the processes that lead to the degradation of ATP to AMP, its dephosphorylation to adenosine, and its subsequent degradation to inosine and hypoxanthine (Table 4(b)).

The energy deficit of CoQ_10_ (group 2, Table 4(a)) reduces the CSF levels of neuroprotective adenosine due to increased degradation of adenosine to inosine and hypoxanthine. Moreover, coenzyme Q_10_ is one of the most important lipophilic antioxidants, preventing the generation of free radicals as well as oxidative modifications of proteins, lipids, and DNA and can also regenerate the other powerful lipophilic antioxidant, *α*-tocopherol. Decreased levels of CoQ_10_ in humans are observed in many pathological conditions (e.g., cardiac disorders, neurodegenerative diseases, AIDS, and cancer) associated with intensive generation of free radicals and their action on cells and tissues [29]. A crucial role in all these processes is played by NAD(P)H-dependent reductase(s) acting as the plasma membrane to regenerate the reduced ubiquinol form of CoQ_10_, contributing to the maintenance of its antioxidant properties [30]. Moreover, correlations of serum levels of Coenzyme Q_10_ and vitamin E isomers (particularly with *γ*-tocopherol) in MS patients show that CoQ_10_ is involved to the mechanisms that lead to alteration in the purine nucleotides metabolism, as well as in the processes of regeneration of vitamin E (Figure 2).

Serum *α*-tocopherol levels were in MS patients within the physiological values, but serum *γ*-tocopherol levels were reduced. Up to 71.4% of MS patients had subliminal serum values of *γ*T and the rest of the MS patients were in the bottom border of the *γ*T physiological values (Figure 1). Serum levels of *γ*T significantly correlated with levels of *α*-tocopherol (*r* = 0.314, *P* = 0.04) (Figure 2).

Vitamin E collectively refers to 8 different structurally related tocopherols and tocotrienols that all possess antioxidant activity. The antioxidant activity of vitamin E is derived primarily from *α*T and *γ*T, of which *α*T is the most biologically active and the predominant form found in blood. During lipid oxidation the isoforms of vitamin E scavenge reactive oxygen species (ROS). This reaction produces oxidized tocopheroxyl radicals that can be recycled back to the active reduced form through reduction by vitamin C. Without reduction of vitamin E by vitamin C, vitamin E can act as ROS donor [31].

In addition to scavenging ROS, *γ*T in contrast to *α*T also react with nitrogen species such as peroxynitrite, forming 5-nitro-*γ*-tocopherol [32]. It has been accepted that the molecules of peroxynitrite are the final molecules responsible for pathological processes in neurodegenerative diseases and MS [33]; 3-nitrotyrosine is an indicator of increased formation of peroxynitrite and the most important molecule considered to be in charge of demyelination [33]. Significantly increased plasma levels of 3-nitrotyrosine were reported in MS patients [15, 34]. Low levels of *γ*-tocopherol and *γ*/*α*-tocopherol ratio in our MS patients might indirectly point out to its high consumption through quenching reactive nitrogen species.

Serum levels of *γ*T showed positive significant relationship between CoQ_10_ (*r* = 0.355,  *P* = 0.02) and *γ*T and IgG index (*r* = 0.314,  *P* = 0.04), indicator of intrathecal synthesis IgG. While both tocopherols exhibit anti-inflammatory activity* in vitro* and* in vivo*, supplementation with mixed (*γ*-enriched) tocopherols seems to be more potent than supplementation with *α*-tocopherol alone [35]. Cook-Mills [36] reported that supplementation with physiological levels of purified natural forms of the vitamin E isoforms *α*T and *γ*T has opposing regulatory functions during inflammation such that *α*T is anti-inflammatory and *γ*T is proinflammatory. Positive correlation of *γ*T with IgG index could indicate proinflammatory effect of *γ*T in MS patients. The imbalance of *α*T/*γ*T levels in plasma may have significant health consequences.

Out of the measured antioxidants, *γ*T correlated the most with CSF levels of purine nucleotide degradation products (Figure 3). Serum levels of *γ*T correlated positively with CSF levels of adenosine, hypoxanthine, xanthine, and metabolic turnover of adenosine to inosine and negatively with CSF levels of uric acid and metabolic turnover hypoxanthine to uric acid (Figure 3).

Mechanism of *γ*T influence on the metabolism of purine nucleotides is not yet known. Participation of *γ*T in the process of purine nucleotides degradation is probably connected with its ability effectively to scavenge NO and other free nitrogen radicals and with scavenging activity of uric acid, which effectively scavenges peroxynitrite formed by the reaction of NO with superoxide [37]. Due to its significant correlation with CoQ_10_, *β*-carotene, and IgG index, it can be assumed that its effect on the metabolism of purine nucleotides has more complex (synergistic) nature.

Levels of *β*-carotene in serum (0.64 ± 0.48 (0.27–1.06) *μ*mol/L) in MS patients were in the lower range of the reference value (0.3–3.0 *μ*mol/L). We observed deficit status of *β*-carotene at approximately 30% of MS patients (Table 3). Serum levels of *β*-carotene significantly negatively correlated with serum TBARS levels (*r* = −0.3295, *P* = 0.048) and with indicators of intrathecal synthesis of IgG-IgG index (−0.4653, *P* = 0.0033) and RIG (*r* = −0.5038, *P* = 0.0017) (Figure 2). After the division of MS patients, according to their vitamin deficiency status into 4 groups (Table 4), MS patients with *β*-carotene deficiency (group 3) in comparison to patients with *γ*T deficiency (group 1) and CoQ_10_ deficiency (group 2) had significantly higher values of IgG index and RIG and in comparison to group 1 (−*γ*T) they had higher levels of TBARS (Table 4(a)).

These results show that *β*-carotene in MS patients participates significantly in the neutralization of lipid peroxidation processes running in this disease. Among the analyzed serum antioxidants, *β*-carotene correlated with TBARS levels only. Beta-carotene in comparison to *α*-tocopherol is more lipophilic and quenches radicals in lipophilic compartments more effective than *α*-tocopherol [38].

Carotenoids are best known for their antioxidant activities including quenching-free radicals, reducing damage from reactive oxidant species, and inhibiting lipid peroxidation. Carotenoids also facilitate cell-to-cell communication which regulates cell growth, differentiation, and apoptosis, and some carotenoids convert to vitamin A [39]. Carotenoids play a pivotal role in prevention of many degenerative diseases mediated by oxidative stress including neurodegenerative diseases [40].

Low levels of *β*-carotene observed in our group of MS patients may be due to a degradation of *β*-carotene in its scavenging activity. It was found that, during the oxidation attacks, carotenoid breakdown products are formed (CBPs), including highly reactive aldehydes and epoxies [41]. Stimulated neutrophils are able to breakdown the *β*-carotene and form a number of CBPs, which inhibit the mitochondrial respiration. This is accompanied by a reduction in the protein sulfhydryl content, a reduction of glutathione (GSH) levels and redox status, and increased accumulation of malonydialdehyde (MDA). Changes in the mitochondrial membrane potential can lead to deterioration in function of adenine nucleotides translocator [42].

Beta-carotene also has anti-inflammatory effects. An inflammatory stimulus, such as IFN-*γ*, activates macrophages to produce various proinflammatory cytokines (TNF*α*, IL-1*β*) and inflammatory mediators, which are synthesized by cyclooxygenase (PGE2) and by induced NO synthase. Expression of these cytokines and genes may be regulated by activation of transcription factor NF-*κ*B. Beta-carotene acts as an inhibitor of redox activation of the transcription factor [42]. The association between inflammation and a decrease in *β*-carotene also showed Van Herpen-Broekmans [43], who found a negative correlation between serum levels of *β*-carotene and inflammatory marker CRP.

## 5. Conclusions

The results of the work show that patients with multiple sclerosis in the early stage of the disease are characterized by reduced antioxidant, immunoregulatory, and neuroprotective ability, which are reflected by the increased metabolism of purine nucleotides, reduced CSF adenosine levels, low serum levels of lipophilic antioxidants *γ*-tocopherol, *β*-carotene, CoQ_10_, and elevated levels of serum TBARS. Serum levels of CoQ_10_ (an indicator of bioenergetic state) and *γ*T (isomer of vitamin E) significantly interfere with the metabolism of purine nucleotides in CSF, while *β*-carotene is rather associated with intrathecal synthesis of IgG and with neutralization of lipid peroxidation processes running in this disease. Due to the fact that one of the possible causes of the axonal degeneration and disability may be an energy deficiency by increased energy requirements for axonal remyelination, demyelination and lipid peroxidation disturbance caused by free radicals production, decreased serum levels of CoQ_10_, and lipophilic antioxidants should be taken into account in clinical practice.

## Figures and Tables

**Table 1:** Levels of selected biochemical parameters in patients with multiple sclerosis (MS) and the neurological control group.

	Reference	Control (*n* = 34)	MS (*n* = 42)	*P* value
Age		36.06 ± 11.92	36.49 ± 11.97	NS
Gender (male : female)		8 : 26	10 : 32	
QA	≤7.4	4.56 ± 1.28	5.37 ± 1.75	0.0293
IgG, CSF (g/L)	0–0.04	0.02 ± 0.01	0.06 ± 0.02	0.0000
IgG, serum (g/L)	7–17	9.32 ± 2.37	11.79 ± 2.58	0.0001
Albumin, CSF (g/L)	0–0.35	0.20 ± 0.07	0.24 ± 0.08	0.0274
Albumin, serum (g/L)	37–53	42.00 ± 3.00	43.04 ± 3.11	NS
IgG index	<0.66	0.44 ± 0.04	0.99 ± 0.36	0.0000
RIG (mg/L)	0	0	14.38 ± 14.76	0.0000

The mean ± standard deviation is shown.

**Table 2:** CSF levels of purine nucleotide degradation products in patients with multiple sclerosis (MS) and in the control group.

	Control (*n* = 34)	MS (*n* = 42)	*P* value
Uric acid (*μ*mol/L)	16.13 ± 3.55	20.45 ± 7.20	0.0029
Hypoxanthine (*μ*mol/L)	2.39 ± 0.84	3.44 ± 1.68	0.0015
Xanthine (*μ*mol/L)	1.67 ± 0.62	2.52 ± 1.02	0.0001
Inosine (*μ*mol/L)	0.91 ± 0.35	1.30 ± 0.40	0.0001
Adenosine (*μ*mol/L)	0.44 ± 0.20	0.20 ± 0.16	0.0000

The mean ± standard deviation is shown.

**Table 3:** Serum levels of some lipophilic antioxidants and TBARS (thiobarbituric acid reactive substances) in patients with multiple sclerosis (MS) compared with reference values.

	Reference values	MS (*n* = 42)	Outside the reference range
*γ*-tocopherol (*μ*mol/L)	2–7	1.54 ± 0.56	71.4% 30/42
*α*-tocopherol (*μ*mol/L)	15–40	22.84 ± 5.65	0% 0/42
*γ*/*α*-tocopherol	0.133–0.175	0.071 ± 0.025	100% 42/42
*β*-carotene (*μ*mol/L)	0.3–3.0	0.64 ± 0.48	28.6% 12/42
Coenzyme Q_10_ (*μ*mol/L)	0.4–1.0	0.43 ± 0.12	45.2% 19/42
TBARS (*μ*mol/L)	<4.5	5.15 ± 0.83	80.9% 34/42

The mean ± standard deviation is shown.

**Table:** (a)

	All MS (*n* = 42)	Group 1 −*γ*T (*n* = 16)	Group 2 −*γ*TCoQ_10_ (*n* = 16)	Group 3 −*γ*T*β*C (*n* = 10)	Group 4 −*γ*TCoQ_10_ *β*C (*n* = 10)
Age	36 (28–40)	37.5 (32–50)	35 (24–37)	37.5 (29–50)	35 (31–38)

QA	4.95 (3.96–6.47)	4.86 (3.45–7.30)	4.69 (4.02–6.53)	5.24 (4.22–8.28)	6.08 (3.96–7.76)

IgG index	0.82 (0.74–1.14)	0.86 (0.72–1.62)	0.79 (0.72–1.25)	1.09 (0.89–1.19)	1.09 (0.72–1.19)

RIG	8.54 (2.43–19.53)	5.40 (2.20–20.67)	5.40 (2.06–8.91)	19.38^∗∗b^ (12.25–20.70)	13.61^∗b^ (4.81–30.46)

*γ*T (*μ*mol/L)	1.50 (1.04–2.00)	1.82 (1.38–2.19)	1.46 (1.29–2.00)	1.70 (1.61–2.11)	1.43 (1.00–1.79)

*α*T (*μ*mol/L)	21.94 (18.65–25.25)	22.90 (19.10–24.40)	21.68 (18.65–24.55)	24.65 (19.10–25.10)	20.60 (17.40–26.20)

*γ*/*α*T	0.07 (0.05–0.09)	0.09 (0.06–0.10)	0.07 (0.05–0.09)	0.08 (0.05–0.09)	0.07 (0.07–0.09)

CoQ_10_ (*μ*mol/L)	0.41 (0.37–0.51)	0.51 (0.43–0.58)	0.38^∗∗ac^ (0.33–0.42)	0.55 (0.47–0.59)	0.38^∗∗ac^ (0.34–0.43)

*β*C (*μ*mol/L)	0.43 (0.27–1.06)	0.90 (0.34–1.21)	0.86 (0.52–1.29)	0.31^∗∗ab^ (0.26–0.374)	0.27^∗∗ab^ (0.24–0.27)

TBARS (*μ*mol/L)	5.11 (4.64–5.64)	4.77 (4.13–5.11)	5.04 (4.44–5.23)	5.15 (4.57–6.28)	5.60^∗∗ab^ (5.19–5.86)

The median (interquartile range) is shown.

*P < 0.05, **P < 0.01; ^a^Versus group 1; ^b^Versus group 2; ^c^Versus group 3.

**Table:** (b)

	Control (*n* = 34)	Group 1 −*γ*T (*n* = 16)	Group 2 −*γ*TCoQ_10_ (*n* = 16)	Group 3 −*γ*T*β*C (*n* = 10)	Group 4 −*γ*TCoQ_10_ *β*C (*n* = 10)
UA (*μ*mol/L)	15.07 (14.12–16.02)	17.96 (12.78–24.11)	21.29 (12.78–23.28)	20.09* (17.88–27.54)	17.06 (16.08–20.62)

Hyp (*μ*mol/L)	2.39 (1.85–3.25)	2.79 (1.98–4.04)	3.23 (2.57–4.59)	2.21 (1.91–3.15)	2.72 (1.91–3.68)

Xan (*μ*mol/L)	1.78 (1.11–2.01)	2.34* (1.54–3.07)	2.39** (1.54–3.24)	1.88 (1.68–2.34)	2.06 (1.88–2.59)

Ino (*μ*mol/L)	0.91 (0.66–1.16)	1.07* (0.94–1.48)	1.19** (1.07–1.53)	1.19** (0.95–1.68)	1.15* (1.10–1.35)

Ado (*μ*mol/L)	0.40 (0.27–0.55)	0.17* (0.13–0.54)	0.12^a∗∗^ (0.07–0.17)	0.17** (0.13–0.24)	0.19** (0.11–0.28)

Ino/Ado	2.02 (1.14–2.43)	5.08** (2.52–7.98)	7.25** (4.92–12.98)	5.35** (3.63–7.98)	6.77** (5.07–9.41)

Hyp/Ino	2.50 (2.15–3.32)	2.60 (1.93–3.31)	2.66 (1.94–3.33)	2.14 (1.57–2.32)	2.87 (1.41–3.23)

Hyp/Ado	5.68 (3.70–7.47)	14.52* (6.54–18.51)	15.72^b,c∗∗^ (14.36–47.5)	14.68* (5.10–17.70)	19.24* (5.10–30.37)

Xan/Hyp	0.67 (0.60–0.75)	0.84 (0.62–0.98)	0.72 (0.63–0.84)	0.88* (0.69–0.99)	0.74 (0.62–0.96)

UA/Xan	9.74 (7.06–12.64)	7.86 (6.57–9.41)	7.58 (6.40–9.41)	7.81 (7.11–9.08)	7.41 (6.15–8.50)

UA/Hyp	6.94 (4.73–7.85)	7.60 (4.51–8.84)	5.79 (4.41–8.55)	7.68 (5.91–8.95)	5.27 (4.69–7.04)

The median (interquartile range) is shown.

*P < 0.05, **P < 0.01 versus control group.

^a^ *P* = 0.0283 versus group 1; ^b^ *P* = 0.0435 versus group 1; ^c^ *P* = 0.0783 versus group 3.
